# Spinal lesions in axial spondyloarthritis: longitudinal assessment of disease-related and degenerative changes

**DOI:** 10.1093/rheumatology/keag153

**Published:** 2026-03-31

**Authors:** Halise Hande Gezer

**Affiliations:** Rheumatology Division, Physical Medicine and Rehabilitation Department, Marmara University School of Medicine, İstanbul, Turkey

**This editorial refers to ‘Relationship between spinal lesions related to axial spondyloarthritis and degenerative spinal lesions: 10-year follow-up of the DESIR cohort’, by Vegas LP et al., 2026; 65(1): keaf694.** 

Structural changes in the spines of patients with axial spondyloarthritis (axSpA) are frequently interpreted within a disease-related framework encompassing both inflammatory and structural processes; however, degenerative lesions are also commonly observed, raising uncertainty about how these processes relate within the same anatomical region. In a study by Vegas et al., published in Rheumatology, a longitudinal perspective is presented to examine whether axSpA-related and degenerative spinal lesions truly influence each other over time [1]. The authors conducted an imaging analysis using detailed 5- and 10-year follow-up data, in which inflammatory and structural lesions related to axSpA were evaluated separately from degenerative spinal changes. The data were derived from the DESIR (DEvenir des Spondyloarthrites Indifférenciées Récentes) cohort, a prospective study of patients with early inflammatory back pain suggestive of axSpA. This study design offers an opportunity to observe the changes in spinal lesions over time and to examine potential relationships between inflammatory and degenerative changes [2].

Degenerative spinal lesions were common initially and were also more frequent at the end of the 10-year follow-up period. On radiographs, the proportion of patients with osteophytes increased from 21% at baseline to 43% at year 10, while the proportion with disc height loss increased from 47% to 65%. Similarly, on MRI, disc degeneration was present in 86% of patients at baseline and in 96% after 10 years of follow-up. Despite this increase in degenerative lesions over time, their appearance and progression could not be explained by axSpA-related lesions or by disease activity measures such as Ankylosing Spondylitis Disease Activity Score (ASDAS). These data are consistent with the possibility that degenerative and axSpA-related inflammatory and structural processes may develop in parallel but largely independently. Although degenerative and axSpA-related spinal lesions in this cohort did not appear to be directly related in the long term, the overall burden of degenerative changes appears notable for a population with a mean age in the mid-30s. Data from general population studies indicate that disk degeneration is observed in asymptomatic individuals at ∼52% at age 30 and 68% at age 40. In comparison, disk height loss is reported at ∼34% and 45%, respectively. Facet joint degeneration, another characteristic of spinal degeneration, is seen in ∼9% of individuals aged 30 and 18% of individuals aged 40 [3]. In contrast, although the degenerative features observed in this axial SpA cohort appear numerically higher, direct comparison is limited by differences in imaging methods and assessment approaches. These observations raise the possibility that degenerative changes in axSpA may not simply reflect normal aging, underlining the importance of future studies that are age- and sex-matched to control populations. In this relatively young cohort, whether the higher burden of degenerative features reflects disease-specific factors, altered biomechanics or increased imaging sensitivity remains an open question.

Although this study evaluates spinal lesions using radiography and MRI, the inflammation detected on imaging does not necessarily reflect the complete histopathological inflammatory activity. Previous studies have shown that tissue-level inflammation can persist even in the absence of MRI involvement, suggesting that conventional imaging methods may miss subclinical inflammation [4]. This limitation should be considered when interpreting the absence of a longitudinal relationship between MRI-detected axSpA-related lesions and degenerative lesions. While a direct association cannot be established, axSpA-related processes may partially contribute to the gradual accumulation of degenerative changes over time.

In addition to biological and imaging considerations, the methodological strategy used to distinguish syndesmophytes from osteophytes represents a key aspect of the study. By excluding spinal units with overlapping features and relying on analyses at adjacent levels, this approach aims to reduce misclassification and enhance internal validity. However, it assumes that inflammatory and degenerative lesions do not develop at the same spinal level, which may not completely reflect the complex biomechanical and biological environment of the spine. Local mechanical loading and tissue responses can differ within a single motion segment. In routine clinical practice, patients may present with both lesion types at the same spinal level, and such mixed patterns may represent important aspects of disease biology that are difficult to capture within a strictly separated analytical scheme [5–7]. By focusing on adjacent vertebral levels rather than direct lesion coexistence, the analysis therefore evaluates indirect spatial relationships, which may underestimate more localized biological interactions. Comparable challenges have been noted in longitudinal imaging studies exploring the complex link between inflammation and new bone formation in axial SpA. This underscores the ongoing challenge of distinguishing mechanical from inflammatory contributions to structural change using currently available imaging definitions.

Treatment exposure represents another potential source of residual confounding over 10 years. However, the analyses accounted for biological disease-modifying antirheumatic drugs use as a time-varying covariate; other therapies such as nonsteroidal anti-inflammatory drugs and glucocorticoids, commonly used in this population, may still influence symptom-driven imaging trajectories and structural outcomes. In addition, adjustments for body mass index were based on baseline values, and changes in body weight over a decade may not be fully captured, despite their potential influence on spinal mechanical loading and degenerative changes.

In conclusion, this study offers valuable long-term insights into the relationship between axSpA-related and degenerative spinal lesions, addressing a question that has rarely been explored using extended longitudinal data. The findings suggest that these processes may evolve in parallel without clear evidence of direct interaction. Future longitudinal studies integrating advanced imaging, biomechanical assessment and molecular markers may further clarify the nature of this relationship.

## Data Availability

No new data were generated or analysed in support of this research.
